# Peripheral neuropathy in patients with multiple sclerosis

**DOI:** 10.1371/journal.pone.0193270

**Published:** 2018-03-07

**Authors:** Adnan Khan, Saadat Kamran, Georgios Ponirakis, Naveed Akhtar, Rabia Khan, Pooja George, Blessy M. Babu, Faiza M. Ibrahim, Ioannis N. Petropoulos, Beatriz G. Canibano, Stacy S. Wilins, Dirk Deleu, Ashfaq Shuaib, Rayaz A. Malik

**Affiliations:** 1 Weill Cornell Medicine-Qatar, Qatar Foundation, Education City, Doha, Qatar; 2 Institute of Neuroscience, Hamad General Hospital, Doha, Qatar; 3 Department of Neurology, University of Alberta, Edmonton, Alberta, Canada; James Cook University Hospital, UNITED KINGDOM

## Abstract

**Objectives:**

To determine the prevalence and severity of neuropathic pain, sudomotor dysfunction and abnormal vibration perception in patients with MS.

**Methods:**

73 patients with MS and 32 age-matched healthy controls underwent assessment of expanded disability severity score (EDSS), DN4 to assess neuropathic pain, electrochemical skin conductance (ESC) to assess sudomotor function and vibration perception threshold (VPT).

**Results:**

Patients with MS had a higher DN4 score (*p* < 0.001) with 14% fulfilling the criteria for neuropathic pain elevated VPT (*p* < 0.001) and lower ESC on the feet (*p* < 0.001) and hands (*p* < 0.001) compared to control participants. ESC on the feet (32% of MS patients) and hands (30% of MS patients) were lower, and DN4 (77% of MS patients) and VPT (64% of MS patients) were greater than 2SD of the healthy control values, respectively. EDSS correlated with the number of relapses (r = 0.564, *p* < 0.001), VPT (r = –0.457, < 0.001) and ESC on the feet (r = –0.268, *p* = 0.023).

**Conclusions:**

Patients with multiple sclerosis have evidence of sudomotor dysfunction and elevated vibration perception, which were associated with neurological disability from MS.

## Introduction

Multiple sclerosis (MS) is considered to be a progressive inflammatory disease characterized by demyelination in the central nervous system including the optic nerves. However, we and others have recently demonstrated significant small fibre damage in patients with MS [1–3]. A recent study has also demonstrated a significant reduction in intraepidermal nerve fibre density in patients with MS [4]. Sudomotor dysfunction has been described in clinically isolated syndrome [5] and abnormal sweating is a feature of MS [6]. Impaired sudomotor function correlates with the severity of clinical disability in MS [7, 8] and is associated with sweat gland denervation [9]. Patients with MS also exhibit the Uhthoff phenomenon, characterized by a worsening of neurological deficits when patients are exposed to heat, suggestive of vasomotor dysfunction.

Sudomotor dysfunction can be quantified using the thermoregulatory sweat test (TST), sympathetic skin response (SSR) or quantitative sudomotor axon reflex test (QSART) [10]. Sudoscan™ is a simple non-invasive technique that quantifies sudomotor function by measurement of electrochemical sweat conductance (ESC) on the hands and feet and has been used to study diabetic neuropathy [11] and more recently transthyretin familial amyloid polyneuropathy [12] and Fabry disease [13].

Abnormalities in thermal and vibration sensation were shown to be associated with impaired somatosensory evoked potentials in patients with MS many years ago [14]. Interestingly, a recent study has shown that patients at increased risk of MS have an elevated vibration perception threshold, even though MRI and OCT are still normal [15]. A recent study has also demonstrated significant abnormalities in proprioceptive, tactile and vibration perception, with altered balance in patients with MS [16]. Moreover, vibration perception threshold was found to be greater in MS patients with walking disability and was related to the 6 min walk test, the Timed Up and Go test and the Berg balance scale [17]. Hand sensation assessed using the Semmes-Weinstein monofilament, duration of vibration with 128-Hz frequency tuning fork, and distance of two-point discrimination has been associated with upper extremity strength and function in patients with MS [18]. Impaired vibration perception correlates with EDSS in relapsing-remitting MS, but not progressive MS [19].

Neuropathic pain in patients with MS has been attributed to central abnormalities and yet MS patients with neuropathic pain have significant abnormalities in thermal thresholds [20]. Indeed, a recent experimental study has demonstrated significant injury to peripheral sensory neurons in autoimmune encephalomyelitis, an experimental model of MS [21]. Furthermore, in a large and detailed phenotyping study of 302 patients with MS, 14% had neuropathic pain according to the DN4 and this was associated with more severe MS and abnormal laser evoked potentials [22].

Thus, a substantial body of data suggests that there is evidence of peripheral neuropathy, particularly, small fibre dysfunction in patients with MS. We have undertaken a detailed evaluation of neuropathic pain, sudomotor function and vibration perception in relation to the type and severity of MS.

## Research design and methods

### Selection of patients

73 patients with MS attending the neurology outpatient clinic at Hamad General Hospital in Doha, Qatar and 32 healthy age and gender-matched controls were studied. This study adhered to the tenets of the declaration of Helsinki and was approved by the Institutional Review Board of Weill Cornell Medicine-Qatar (approval no. 15–00064). Informed, written consent was obtained from all participants before participation in the study. Patients with a diagnosis of MS (fulfilling the 2010 revised McDonald Criteria) and aged 18–65 were included in the study. Patients were classified into those with clinically isolated syndrome (CIS, n = 8), relapsing-remitting MS (RRMS, n = 48) and secondary progressive MS (SPMS, n = 19), based on the clinical course. Other causes of peripheral neuropathy (family history, diabetes, vitamin B12/folate deficiency, electrophoresis, ANA, alcohol excess) were excluded. Participants on medication, which could affect sudomotor function, were excluded.

### Neurological evaluation

The neurological status of subjects with MS was assessed using the expanded disability status scale (EDSS). Disease duration was calculated as years from onset to the last assessment of disability. A Timed 25-foot Walk Test (T25FW), Nine- Hole Peg Test (9-HPT) and the oral Symbol Digit Modality Test (SDMT) were performed in all participants.

The DN4-interview questionnaire (0–10) was used to identify neuropathic pain [23]. Vibration perception threshold (VPT) was assessed using a Neurothesiometer (Horwell; Scientific Laboratory Supplies, Wilfrod, Nottingham, U.K.) with an average of three consecutive readings on the great toe for each foot. Sudoscan™ (Impeto Medical, Paris, France) was used to measure sudomotor function [24]. The test was performed in a temperature controlled room with a room temperature of 25 ± 2 C°. For the Sudoscan assessment, patients were advised to simultaneously place their bare hands and feet on two sets of large-area nickel electrode plates for 3 minutes without movement. The electrochemical skin conductance (ESC), expressed in microSiemens (μS), is the ratio between the current generated and the constant DC stimulus (≤4 V) applied to the electrodes. At low voltages (<10 V), the stratum corneum is electrically insulating and only sweat glands are conductive.

## Statistical analysis

All statistical analyses were performed using SPSS and graphic illustrations were generated using Prism 6 (version 6.0g for Window, Graphpad software Inc., CA, USA). Normality of the distribution of data was examined using the Kolmogorov-Smirnov test and by visual inspection of histogram and normal Q-Q plot. Data is expressed as the mean ± standard deviation (Table 1). The data used for statistical analysis in this study are available on (https://figshare.com/s/9a7938564b35bd9b4cb6).

**Table 1:** Demographic and neurological assessment in Healthy Controls (HC) compared to patients with multiple sclerosis (MS) and also subdivided into those with CIS, RRMS and SPMS.

Parameters (number of patients)	HC (n = 32)	MS (n = 73)	CIS (n = 8)	RRMS (n = 46)	SPMS (n = 19)
Age (years)	33.29 ± 4.49	36.68 ± 9.44	36.63 ± 7.27	34.74 ± 8.68	39.84 ± 9.18
Gender (M/F)	13/19	25/48	3/5	14/32	8/11
MS Duration (years)^**^	–	7.72 ± 3.97	4.00 ± 2.94	7.59 ± 3.55	9.73 ± 4.08^**§**^
Relapses (number)^**^	–	1.82 ± 2.09	–	1.08 ± 0.93	3.75 ± 2.67^**¥**^
EDSS (score)^***^	–	1.49 ± 1.82	0.75 ± 0.65	0.73 ± 0.93	3.63 ± 2.11^**§**^^,^^**¥**^
Symbol Digit Modality Test (score)^***^	52.32 ± 8.37	40.23 ± 13.19^**†**^	44.40 ± 9.76	42.53 ± 12.35^**‡**^	33.14 ± 14.31^**‡**^
25 Foot Walk Test^***^	3.50 ± 0.62	7.29 ± 4.19^**†**^	6.20 ± 1.18^**‡**^	6.41 ± 2.68^**‡**^	10.16 ± 6.71^**‡**^
9-HP Test (seconds)	18.58 ± 2.35	23.90 ± 5.82^**†**^	22.38 ± 1.34^**‡**^	22.85 ± 5.39^**¥**^	26.97 ± 7.11^**‡**^
**Peripheral Neuropathy Assessment**					
Parameters (number of patients)	HC (n = 32)	MS (n = 73)	CIS (n = 8)	RRMS (n = 46)	SPMS (n = 19)
VPT^***^	3.55 ± 1.32	9.64 ± 8.20^**†**^	12.46 ± 10.26	7.77 ± 7.15^**‡**^	13.07 ± 8.99 ^**‡**^
Feet ESC (μS)^***^	79.23 ± 5.90	69.08 ± 17.47^**†**^	66.63 ± 22.51	73.28 ± 13.79	59.33 ± 20.72^**‡**^
Hands ESC (μS)^*^	72.30 ± 8.31	62.10 ± 17.14^**†**^	59.00 ± 19.85	64.87 ± 15.63^**‡**^	55.89 ±19.11^**‡**^
DN4^***^	0.09 ± 0.30	1.83 ± 1.80^**†**^	1.75 ± 1.58	1.76 ± 1.87^**‡**^	2.05 ± 1.78^**‡**^

Results are expressed as mean ± SD. Statistically significant differences between different types of MS groups using ANOVA

^*^ *p* < 0.05

^**^ *p* < 0.01

^***^ *p* < 0.001.

^**‡**^ Post hoc results differ significantly from healthy control group (*p* < 0.05).

^**§**^Post hoc results differ significantly from CIS (*p* < 0.05).

^**¥**^ Post hoc results differ significantly from RRMS (*p* < 0.05).

^**†**^ Results differ significantly from healthy controls (*p* < 0.001).

To assess within and between-group differences, we used one-way ANOVA (non-parametric, Kruskal-Wallis test) and Tundden T3 for posthoc test corrections. Chi-square (X^2^) test for demographic variables. Student t-test was used to compare healthy controls to patients with MS. Spearman rank test was used and expressed as a correlation coefficient (r) to estimate the strength of the relationship between clinical disability (EDSS) and neuropathy assessment. P < 0.05 was considered to be significant.

## Results

The clinical, demographic and peripheral neuropathy assessment parameters of the participants in the study are given in Table 1.

### Clinical, neurological and peripheral neuropathy assessment

MS patients and healthy control (HC) participants were age-matched and there were no significant demographic differences between the two groups. Patients with MS had a disease duration of 7.72 ± 3.97 years with 1.82 ± 2.09 relapses and an EDSS of 1.49 ± 1.82. They had a significant reduction in the SDMT score (40.23 ± 13.19 vs 52.32 ± 8.37, *p* < 0.001), longer T25FW (7.29 ± 4.19 vs 3.50 ± 0.62, *p* < 0.001) and 9-HPT (23.90 ± 5.82 vs 18.58 ± 2.35, *p* < 0.001) compared with control participants.

**Fig 1:**
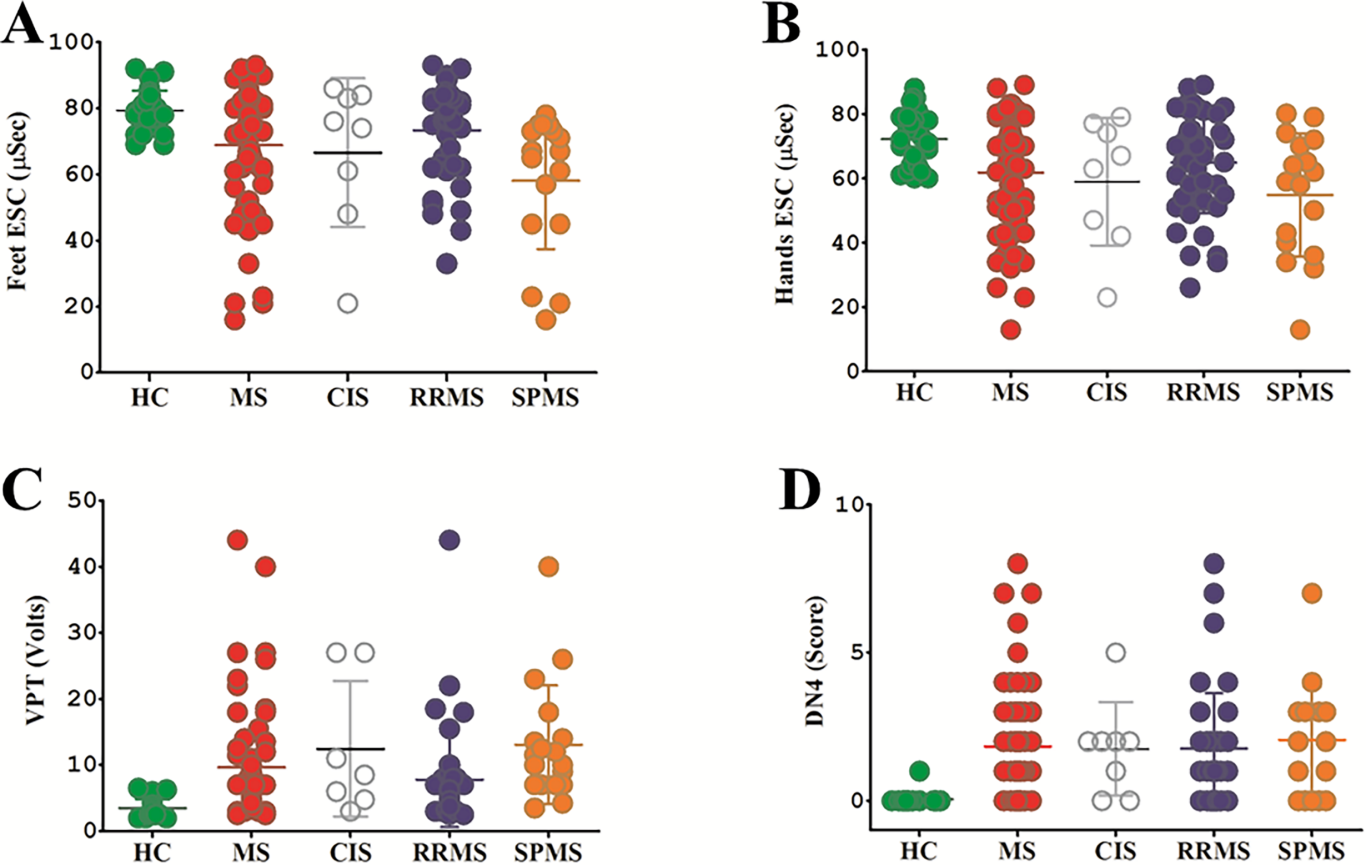
**ESC in the feet (A) and hands (B), VPT (C) and DN4 (D) in healthy controls compared to patients with MS and according to the sub-type of MS**.

Patients with MS had a significantly greater DN4 score (1.83 ± 1.80 vs 0.09 ± 0.30, *p* < 0.001), with 14% fulfilling the criteria for neuropathic pain, lower ESC on the feet (69.08 ± 17.47 vs 79.23 ± 5.90, *p* < 0.001) and hands (62.10 ± 17.14 vs 72.30 ± 8.31, *p* < 0.001) and elevated VPT (9.64 ± 8.20 vs 3.55 ± 1.32, *p* < 0.001) compared to control participants (Fig 1).

ESC on the feet (32% of MS patients) and hands (30% of MS patients) were lower, and DN4 (77% of MS patients) and VPT (64% of MS patients) were greater than 2SD of the healthy controls, respectively.

### Different types of MS

The time from diagnosis of MS was significantly shorter in patients with CIS compared to SPMS (*p* < 0.05). EDSS was significantly higher in SPMS vs CIS (*p* < 0.001) and RRMS (*p* < 0.001). There were no differences in age, gender, SDMT score, T25FW, 9-HPT, VPT, ESC on the hands and feet or DN4 between different MS subtypes.

### Correlation of EDSS and peripheral neuropathy parameters

There was no correlation between EDSS and age, MS duration, SDMT, DN4 and ESC on the hands. However, EDSS correlated with the number of relapses (r = 0.564, *p* < 0.001), T25FW (r = 0.360, *p* = 0.01), VPT (r = –0.457, < 0.001) and ESC on the feet (r = –0.268, *p* = 0.023). There was no correlation between ESC on the feet and the number of relapses, T25FW and 9-HPT, but it did correlate with age (r = –0.405, *p* < 0.001), MS duration (r = –0.387, *p* < 0.004) and SDMT (r = 0.292, *p* = 0.036). There was no correlation between ESC on the hands and the number of relapses, T25FW and 9-HPT, but there was a correlation with MS duration (r = –0.334, *p* = 0.014). VPT correlated with age (r = 0.354, *p* = 0.003) and 9-HPT (r = 0.367, *p* = 0.008) and DN4 correlated with age (r = –0.371, *p* = 0.001).

## Discussion

This study demonstrates an increased prevalence of sudomotor dysfunction and elevated vibration perception in patients with MS. Although the severity of abnormality is mild, a substantial proportion of patients with MS show significant evidence of abnormality in both ESC and VPT and 14% have evidence of neuropathic pain.

This study confirms previous studies showing sudomotor dysfunction in patients with MS [6, 8]. Indeed, an abnormality in QSART has been demonstrated in 32.7% of patients with CIS [25]. We also demonstrate correlations between the different measures of neuropathy and the severity of MS, but no difference between the sub-types of MS. In the present study, we show sudomotor dysfunction using Sudoscan and add MS to the many neurological conditions that this device can be used to identify sudomotor dysfunction and hence a small fibre neuropathy.

Whilst we show a mild elevation in vibration perception threshold, surprisingly this was present in 64% of patients with mild MS and correlated with disease severity. This is in accord with earlier studies showing that 64% had an abnormal vibration perception [26] and that 26% of patients with mild MS had an elevated vibration perception thresholds in the feet [27]. Elevated vibration perception threshold rather than thermal thresholds have also been correlated with somatosensory evoked potentials in patients with MS [28]. Whilst the elevated VPT may be attributed to dorsal column involvement, this would not explain the presence of neuropathic pain and sudomotor dysfunction.

Evoked potentials have been proposed as an objective means to assess disease severity and monitor progression or regression in clinical trials of patients with MS [29]. In a recent study of 34 patients with MS, elevated vibration perception threshold correlated with the 6 minute walk test, Timed Up and Go test and the Berg balance scale [30]. However, in our study, there was no correlation between VPT and the timed 25-foot walk test.

Several studies have reported neuropathic pain in patients with MS [22, 31]. Our study has also demonstrated an identical 14% prevalence of neuropathic pain in patients with MS, but there was no correlation to EDSS [32]. This is in agreement with a recent study that found an increased prevalence of neuropathic pain in MS patients, which was also not associated with EDSS [33]. Indeed, some studies have shown an association between pain and EDSS [34] while others have not [35].

The findings of the present study in patients with MS merit larger longitudinal studies to assess the underlying basis of both small and large fibre neuropathy in relation to disease sub-type, severity and progression.

## Supporting information

S1 File Data of the peripheral neuropathy in patients with multiple sclerosis.xlsx. (ZIP)
